# Effects of calorie labeling and value size pricing on fast food meal choices: Results from an experimental trial

**DOI:** 10.1186/1479-5868-5-63

**Published:** 2008-12-05

**Authors:** Lisa J Harnack, Simone A French, J Michael Oakes, Mary T Story, Robert W Jeffery, Sarah A Rydell

**Affiliations:** 1 Division of Epidemiology and Community Health, School of Public Health, University of Minnesota, Minneapolis, Minnesota, USA

## Abstract

**Background:**

Although point-of-purchase calorie labeling at restaurants has been proposed as a strategy for improving consumer food choices, a limited number of studies have evaluated this approach. Likewise, little research has been conducted to evaluate the influence of value size pricing on restaurant meal choices.

**Methods:**

To examine the effect of point-of-purchase calorie information and value size pricing on fast food meal choices a randomized 2 × 2 factorial experiment was conducted in which participants ordered a fast food meal from one of four menus that varied with respect to whether calorie information was provided and whether value size pricing was used. Study participants included 594 adolescents and adults who regularly ate at fast food restaurants. Study staff recorded the foods ordered and consumed by each participant. Participants also completed surveys to assess attitudes, beliefs and practices related to fast food and nutrition.

**Results:**

No significant differences in the energy composition of meals ordered or eaten were found between menu conditions. The average energy content of meals ordered by those randomized to a menu that included calorie information and did not include value size pricing was 842 kcals compared with 827 kcals for those who ordered their meal from a menu that did not include calorie information but had value size pricing (control menu). Results were similar in most analyses conducted stratified by factors such as age, race and education level.

**Conclusion:**

Additional research is needed to better evaluate the effects of calorie labeling and value size pricing on fast food meal choices. Studies in which participants are repeatedly exposed to these factors are needed since long term exposure may be required for behavior change.

## Background

The prevalence of overweight and obesity in the United States has increased dramatically [1]. One factor that many believe to be an important contributor to this increase is the number of meals and snacks eaten away from home. Over the past several decades the proportion of total food expenditures spent on food away from home has increased from 34% in 1974 to about half in 2004 [2]. Foods available at restaurants and other away from home eating locations tend to be higher in calories and fat [3-6] and often larger in portion size [7,8] compared to foods eaten from home, which may contribute to energy intake in excess of energy expenditure.

In recognition of the potential role of meals eaten away from home on excess energy intake, a number of strategies to promote more healthful food choices when eating out have been proposed [9-13]. One recommended approach is to increase the availability of nutrition information for foods eaten and prepared away from home [9-13]. Moreover, it has been suggested that fast food chain restaurants be required to provide calorie information on their menu boards or on product packaging [10]. In theory, the provision of calorie information at the point-of-purchase for restaurant products may help consumers limit excess calorie intake.

Aside from providing point-of-purchase nutrition information, it has also been suggested that the food industry reduce its use of value size pricing as a marketing technique [12]. Value size pricing involves structuring product prices such that the per unit cost (e.g., price per ounce) decreases as portion size increases. It has been speculated that this product pricing structure leads to higher energy intake when eating out because value conscious consumers may be prone to purchase larger-sized food items [12].

Although a number of studies have been conducted to evaluate the effect of point-of-purchase nutrition promotions on foods purchased away from home [14-37], the relevance of most of these studies to calorie labeling in a restaurant context is limited for a number of reasons. First, many of the studies evaluated point-of-purchase promotional activities that focused solely on identifying and promoting more healthful food choices (e.g., low-fat foods) [16,19-23,27,28,30-33] versus providing nutrient content information, such as calorie information, for the full range of available foods. Many of the studies were conducted in a workplace or university setting [14-20,31-37] instead of a restaurant. Among those studies that evaluated comprehensive calorie labeling (e.g., providing calorie information for most food items) [14,15,17,18,24,26,34-37], a number evaluated self-reported behavioral intentions [14,24-26,29,37] rather than actual food purchases.

In consideration of the limitations of previous studies, we conducted an experimental trial to examine the effects on food purchases of providing calorie information at the point-of-purchase for food items on a fast food restaurant menu. The influence of value size pricing on fast food meal choices and consumption was also examined.

## Methods

### Overview

**Figure 1:**
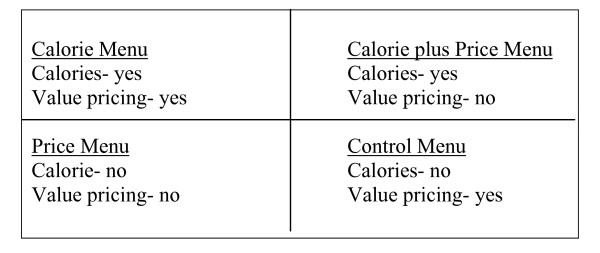
Diagram of 2 × 2 experimental design menus.

A randomized controlled 2 × 2 factorial experiment was conducted in which adolescents and adults who reported eating regularly at fast food restaurants were asked to purchase and consume a fast food restaurant meal from one of four randomly assigned menus. The menus varied as to whether calorie information was provided and value size pricing was used (see Figure 1). Menu items ordered and consumed by each study participant were recorded by trained study staff.

### Study Menus

Four paper menus designed to be similar in format to menu boards at fast food restaurants were developed. Food items on all four menus were lunch and dinner items available at McDonald's at the start of the experiment (October 2005). Thus, the menu included a variety of foods and beverages including hamburgers (n = 9), fish and chicken entrees (n = 6), salads (n = 4), french fries (n = 1), beverages (n = 9), and desserts (n = 4). All of the portion size options available at McDonalds at the time of the study were included on the menu as well. To blind participants to the meal source some food descriptions were modified. For example, the Big Mac™ hamburger was given the generic description 'Double Cheese Burger Deluxe'. Each of the four study menus is briefly described as follows:

#### Calorie Menu

**Figure 2:**
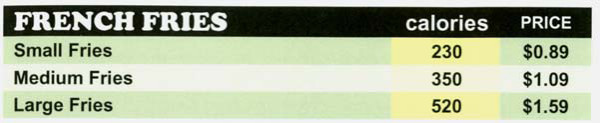
Excerpt from calorie menu.

**Figure 3:**
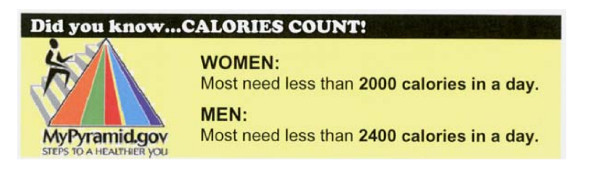
Calorie reference information provided in the bottom right hand corner of the calorie and calorie plus price menus.

The calorie menu included calorie information for each menu item. This information was provided in a column between the food description and product price columns (see Figure 2 for menu excerpt). To draw attention to it, the background color of the calorie column was bright yellow. To put the calorie information in context the average daily calorie needs of adult men and women were provided in a 'Calories Count' information box in the bottom right hand bottom corner of the menu (see Figure 3). The calorie content information for each item was obtained from the McDonald's Corporation web-site. Food items were priced in accord with McDonald's usual food pricing for the area.

#### Price Menu

**Figure 4:**
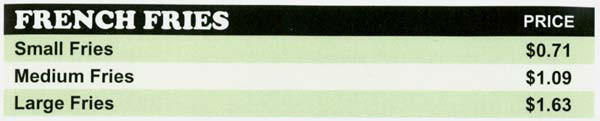
Excerpt from price menu.

The price menu was modified for items with more than one portion size option so that the value size pricing structure was eliminated from the menu. The prices listed for food items that had more than one portion were calculated so that the price per ounce was standardized across portion size options (see Figure 4 for menu excerpt).

To calculate the standardized prices, a standard price per ounce for each product type (e.g., soft drinks) was determined and applied to calculate the total price for each product portion size. The standard price per ounce used in this calculation was based on the price per ounce of the medium-sized portion available (price of medium serving divided by the total number of ounces in a medium serving). Using this price per ounce estimate, the price for each product portion size option was calculated by multiplying the standard price per ounce by the number of ounces in each product portion. The price menu did not include calorie information.

#### Calorie plus Price Menu

**Figure 5:**
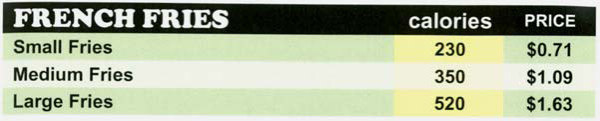
Excerpt from calories plus price menu.

The calorie plus price menu included calorie information and price modification. The calorie information provided was identical to that on the calorie menu. The price modification was identical to that of the price menu (see Figure 5 for menu excerpt).

#### Control Menu

**Figure 6:**
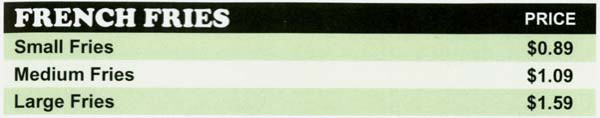
Excerpt from control menu.

The control menu did not include calorie information and all menu items were priced in accord with usual McDonald's pricing, and thus included value size pricing (see Figure 6 for menu excerpt).

#### Participant Recruitment

Participants were recruited from suburban and urban communities in the Minneapolis St. Paul, Minnesota metropolitan area via advertisements placed in community newspapers and flyers posted in community locations. Also, recruitment was conducted in-person at selected area high school. A $25 gift certificate to a discount store was offered as an incentive to participate.

Those who called the recruitment phone number provided in advertisements and flyers were screened for the following eligibility criteria: 1) ≥ 16 years of age; 2) eat at fast food restaurants ≥ 1 time/week; and 3) able to read and speak English. Those who met eligibility criteria were told that participation would involve completing a two-hour evening study session at which they would be required to purchase a fast food restaurant meal for their dinner and complete several questionnaires. To minimize subject reactivity participants were blinded to the menu manipulation aspect of the study. The study purpose was described as "learning more about fast food meal choices". Participants were also blinded to the source of the fast food. Those who indicated an interest in participating were scheduled for a study session, and randomly assigned to one of the four menu conditions.

### Study Session Procedures

Study sessions were held on weekday and weekend evenings (4:50 pm–7:30 pm) between October 2005 and April 2006. The sessions were held in three sites. Two of the sites were conference rooms in suburban hotels. One site was the basement of an urban church. Each location was within six blocks of a McDonald's restaurant.

Upon arrival at the study session each participant met individually with a study staff member who provided him/her with the study menu to which they were randomly assigned. The participant was asked to order their dinner from the menu, and the study staff member recorded the order. After ordering participants were told that payment for their meal would be collected from them at the end of the study session, and then they were escorted to a room set up as a dining area. Participants were asked to complete a survey while waiting for their food. The survey included questions about fast food consumption frequency, opinions about fast food restaurants, and food shopping and preparation practices.

Immediately after participants placed their dinner order study staff drove to the nearby McDonald's restaurant to purchase the meals ordered by participants. Participants were then provided their food by study staff. When participants were finished eating, leftover food was collected and covertly measured using a digital food scale.

After meals were consumed participants were escorted to an exit interview area where a final interview was completed by a research staff person. This interview included questions about nutrition knowledge and beliefs and self-reported height and weight. After participants completed the interview they were informed of the true intent of the study (debriefed) and told that they would not have to pay for their meal. They were then asked several questions to determine whether they had noticed the menu manipulations, and whether they were aware of the true purpose of the study or the source of the food prior to the study session.

The University of Minnesota Institutional Review Board approved all study procedures.

### Determining Nutrient Composition of Meals Selected and Consumed

The nutrient composition of the meals selected and consumed by participants were calculated using a food composition table available from the McDonald's corporation [38] in combination with the gram weight information for the amount of each food item selected and consumed. Although energy (kcal) was the primary focus, estimates for total fat, total carbohydrate, total protein, saturated fat, dietary fiber, vitamin C, and calcium were also generated so that possible experimental effects on selection and consumption of these nutrients could be examined.

### Data Analyses

A total of 605 individuals completed the study procedures. Eleven of these individuals were excluded from the analyses because they disclosed during the debriefing that they knew before participating in the study that calories might be listed or price would be modified on the menu (n = 2) or knew that they would not have to pay for their meal (n = 9).

Means and frequencies were calculated to describe the characteristics of the study sample. To evaluate whether randomization was effective in equally distributing potential confounders across experimental conditions, characteristics of participants such as age, sex, and education level were compared across experimental groups.

General linear modeling (GLM) [39] was conducted to test for differences in the food and nutrient composition of meals selected and consumed by those in each experimental condition. In each model, the food or nutrient under consideration was included as the dependent variable and experimental condition was included as the independent variable. Possible differences in experimental condition effects by age (16–28 years, 29–49 years, 50+ years), sex (male, female), highest education level (college graduate or higher, less than college graduate), body weight (normal weight, overweight or obese), and perceived importance of nutrition and price when eating fast food were examined by conducting analyses stratified by these factors, and qualitatively comparing results. Tests for interactions were not conducted because power for two-way interactions was weak.

All analyses were conducted using SAS (Version 9.1.2, 2004; SAS Institute, Cary, NC).

### Statistical Power

The study was designed to have strong power (85%) to detect small main effects (delta < 100 kcal; effect size = 0.2) under ideal conditions (e.g., low variance, perfect randomization, Gaussian errors). Modest to strong effect sizes may be detected in analyses conducted stratified by factors such as sex and education level. For example, in analyses involving one-half of the sample, modest effects (delta < 140 kcal) are detectable.

## Results

The demographic characteristics of participants are shown in Table 1. More females (59.4%) than males (40.6%) participated. Approximately three-fourths of participants were white, with blacks comprising the second largest racial/ethnic group (10.9%). The demographic characteristics of participants in each experimental group were similar.

**Table 1:** Demographic characteristics of participants by experimental group

	Total (n = 594) % (n)	Calorie^a^ (n = 151) % (n)	Price^b^ (n = 143) % (n)	Calorie + price^c^ (n = 150) % (n)	Control^d^ (n = 150) % (n)	p-value^e^
Age (years)						
16–25	24.8 (147)	19.9 (30)	31.5 (45)	21.3 (32)	26.7 (40)	0.08
26–40	19.4 (115)	14.6 (22)	20.3 (29)	22.0 (33)	20.7 (31)	
41–60	41.8 (248)	46.4 (70)	35.7 (51)	41.3 (62)	43.3 (65)	
≥ 61	14.1 (84)	19.2 (29)	12.6 (18)	15.3 (23)	9.3 (14)	
Sex						
Male	40.6 (241)	37.7 (57)	37.8 (54)	46.0 (69)	40.7 (61)	0.42
Female	59.4 (353)	62.3 (94)	62.2 (89)	54.0 (81)	59.3 (89)	
Ethnicity						
Hispanic/Latino	3.4 (20)	1.3 (2)	5.7 (8)	4.0 (6)	2.7 (4)	0.21
Not Hispanic/Latino	96.6 (567)	98.7 (148)	94.3 (133)	96.0 (144)	97.3 (142)	

Education level^f^						
High school	25.3 (150)	29.1 (44)	23.1 (33)	20.7 (31)	28.2 (42)	0.54
graduate or less	38.8 (230)	39.1 (59)	41.3 (59)	40.0 (60)	34.9 (52)	
Some college	35.9 (213)	31.8 (48)	35.7 (51)	39.3 (59)	36.9 (55)	
College graduate or higher						
Body weight^g^						
Normal weight	42.6 (249)	43.2 (64)	45.1 (64)	40.1 (59)	41.9 (62)	0.83
Overweight	27.9 (163)	27.0 (40)	26.1 (37)	32.7 (48)	25.7 (38)	
Obese	29.6 (173)	29.7 (44)	28.9 (41)	27.2 (40)	32.4 (48)	

^a ^calorie menu included calorie information for each menu item

^b ^price menu had price modification (standardized pricing) for food items with more than one portion size option

^c ^calorie + price menu included calorie information and price modification (standardized pricing) for food items with more than one portion size option.

^d ^control menu did not include calorie information and had usual food pricing

^e ^p-value calculated from chi-square test.

^f ^For participants 16–19 years of age, the reported education level is that of their parent with the highest degree or level of education.

^g ^For those 16–19 years of age: CDC growth charts were used to calculate percentiles for sex and age. In this table, those < 85^th ^percentile were classified as normal weight; 85–94^th ^percentile were classified as overweight; and ≥ 95^th ^percentile were classified as obese; For those ≥ 20 years of age: body mass index < 25 was classified as normal weight; 25–29.9 was classified as overweight; and ≥ 30 was classified as obese.

When asked to rate the importance of price, taste, nutrition and convenience when purchasing food from a fast food restaurant and when buying groceries, taste was the most highly rated factor for both; 97.6% and 98.5% reported taste as very important or somewhat important when buying fast food and groceries, respectively (Table 2). In contrast, nutrition was the least likely to be rated as very important or somewhat important, with 58.2% and 83.5% of participants rating nutrition as very important or somewhat important when buying fast food and groceries respectively.

**Table 2:** Importance of taste, price, nutrition, and convenience when purchasing food from a fast food restaurant and the grocery store

	Very important % (n)	Somewhat important % (n)	Not very important% (n)	Not at all important % (n)
**Fast food**				
Taste	76.9 (456)	20.7 (123)	1.7 (10)	0.7 (4)
Convenience	56.4 (333)	35.4 (209)	6.8 (40)	1.4 (8)
Price	40.4 (239)	43.4 (257)	13.2 (78)	3.0 (18)
Nutrition	20.8 (122)	37.4 (219)	29.4 (172)	12.5 (73)
**Groceries**				
Taste	78.3 (461)	20.2 (119)	1.5 (9)	0 (0)
Convenience	34.5 (202)	47.7 (279)	14.5 (85)	3.3 (19)
Price	59.4 (350)	34.6 (204)	5.3 (31)	0.7 (4)
Nutrition	39.7 (233)	43.8 (257)	11.4 (67)	5.1 (30)

The average energy and nutrient composition of meals ordered and consumed by those in each experimental group (Table 3) were similar. For example, there were no significant differences (p = 0.25) in the average number of calories consumed by those in the calorie, price, calorie plus price, and control menu conditions were 805, 813, 761, and 739 respectively. Selection and consumption of major food categories (e.g., sugar-sweetened soft drinks, diet soft drinks, French fries, salads, etc.) and portion sizes were also examined, with no significant differences found (data not shown).

**Table 3:** Mean nutrient contents of meals ordered and consumed by participants in each experimental group

	Calorie^a^	Price^b^	Calorie + Price^c^	Control^d^	p-value^e^
**Ordered**					
Energy, kcal	873.6 (439.1)	881.7 (353.6)	842.3 (425.3)	827.5 (400.6)	0.62
Total fat, g	34.3 (19.3)	35.1 (15.1)	32.7 (17.0)	32.5 (18.6)	0.55
Total carbohydrate, g	110.3 (63.3)	112.7 (55.8)	108.0 (65.2)	105.7 (39.2)	0.77
Total protein, g	32.4 (14.5)	30.5 (11.3)	30.4 (13.)	29.9 (12.0)	0.37
Saturated fat, g	10.7 (7.6)	10.4 (5.7)	10.3 (6.7)	9.7 (6.7)	0.61
Dietary fiber, g	5.0 (2.8)	5.2 (2.8)	4.8 (2.7)	4.6 (2.9)	0.33
Vitamin C, mg	27.1 (47.1)	24.1 (39.0)	19.9 (32.4)	27.0 (45.2)	0.39
Calcium, mg	314.2 (233.8)	270.7 (190.8)	303.3 (234.7)	272.7 (213.7)	0.22
**Consumed**					
Energy, kcal	804.7 (423.9)	813.3 (331.6)	761.0 (356.8)	739.0 (358.2)	0.25
Total fat, g	32.1 (19.1)	32.8 (15.1)	30.1 (15.3)	29.6 (16.3)	0.29
Total carbohydrate, g	100.0 (58.6)	102.7 (49.8)	96.0 (53.3)	92.0 (52.4)	0.34
Total protein, g	30.4 (14.4)	28.5 (10.2)	28.0 (11.4)	27.8 (11.1)	0.20
Saturated fat, g	9.9 (7.5)	9.8 (5.5)	9.4 (6.0)	8.9 (6.1)	0.50
Dietary fiber, g	4.7 (2.8)	4.8 (2.8)	4.5 (2.6)	4.2 (2.7)	0.22
Vitamin C, mg	26.2 (46.2)	22.5 (36.3)	20.2 (34.3)	24.5 (39.4)	0.60
Calcium, mg	285.1 (215.0)	248.1 (163.7)	265.3 (191.4)	246.1 (191.0)	0.26

^a ^calorie menu included calorie information for each menu item

^b ^price menu had price modification (standardized pricing) for food items with more than one portion size option

^c ^calorie + price menu included calorie information and price modification (standardized pricing) for food items with more than one portion size option.

^d ^control menu did not include calorie information and had usual food pricing

^e ^p-value calculated from an analysis of variance analysis

**Figure 7:**
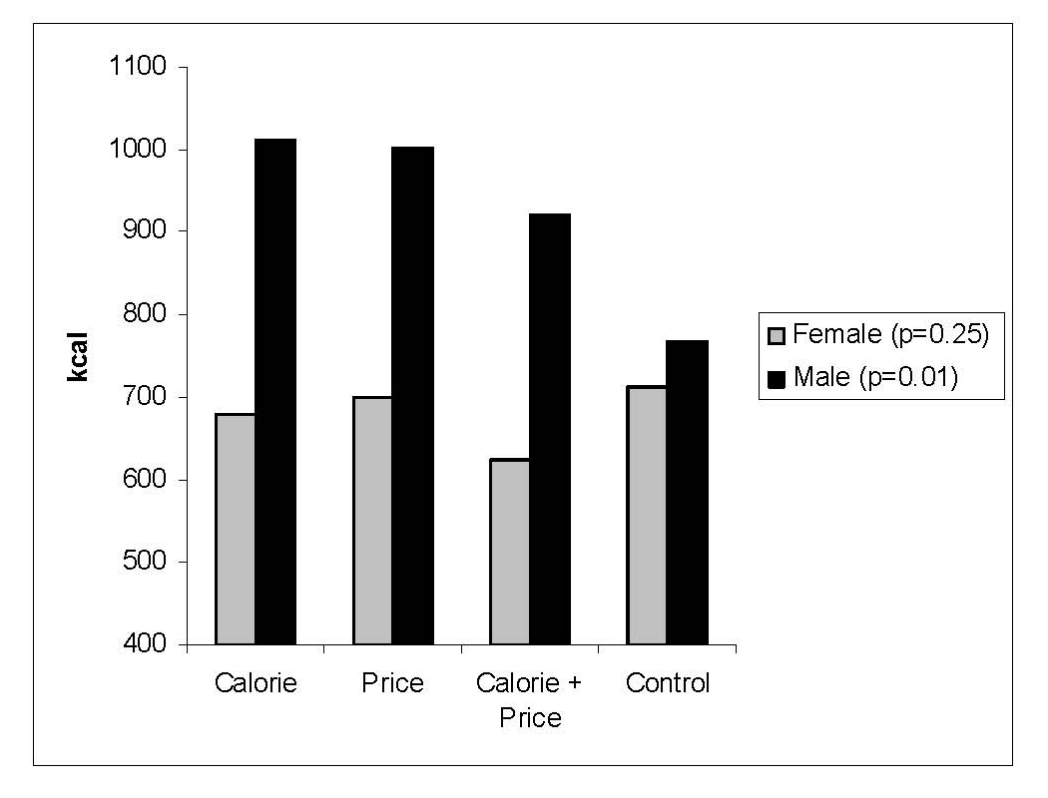
Average energy intake by experimental condition among females (n = 353) and males (n = 241).

**Figure 8:**
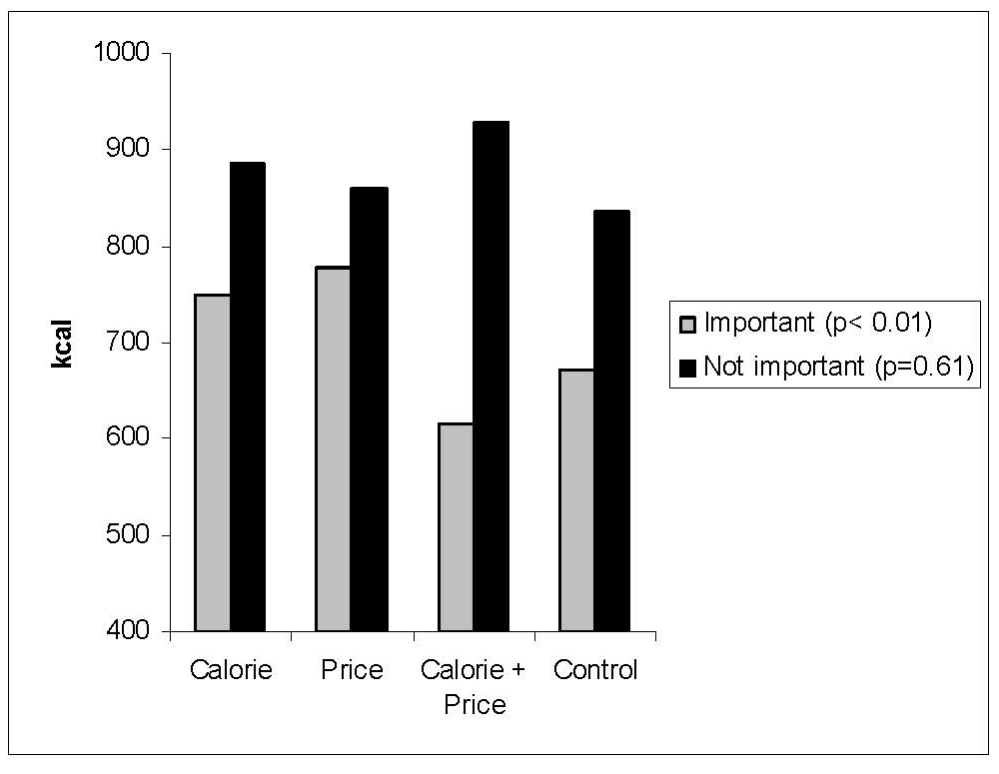
Average energy intake by experimental condition among those who reported nutrition was very important or somewhat important (n = 341) or not very important or not at all important (n = 245) when buying foods from a fast food restaurant.

**Figure 9:**
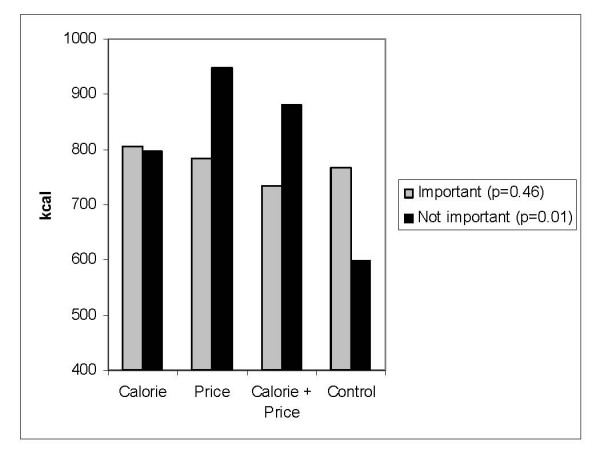
Average energy intake by experimental condition among those who reported price was very important or somewhat important (n = 496) or not very important or not at all important (n = 96) when buying foods from a fast food restaurant.

The average energy content of meals selected and consumed were similar across experimental conditions among those in each age, education level, and body weight strata. However, significant differences in energy intake across experimental condition were observed among males (p = 0.01), those who reported that nutrition was important when buying food from a fast food restaurant (p < 0.01), and those who reported price was not important when buying food from a fast food restaurant (p = 0.01). Average energy intake was higher among males in the calorie, price, and calorie plus price experimental conditions compared to those who selected their meal from the control menu (Figure 7). Among those who reported that nutrition was important when buying fast food, average energy intake was significantly lower among those who received the control and calorie plus price menus relative to those in the other two experimental conditions (Figure 8). Among those who reported that price was not important when buying food from a fast food restaurant, average energy intake was lowest among those in the control condition (598 kcal) and highest among those in the calorie plus price condition (948 kcal) (Figure 9).

As part of the post-meal interview, participants in the calorie, price, and calorie plus price menu conditions were asked questions to assess whether they noticed the menu manipulations. Those who received a menu with calories listed were asked, "Did you notice that calorie counts were listed on your menu this evening?". About half (54%) of those in the calorie condition and 59% of those in the calorie plus price menu conditions reported noticing the calorie information (Table 4). Those with a higher level of education, whites, and those 15–25 years of age were more likely to report noticing the calorie information (data not shown). Participants who received the price or calorie plus price menu were asked, "Did you notice on the menu from which you ordered tonight that prices were set such that for a given unit of food (like a chicken nugget or ounce of soda), you paid the same price for that unit no matter what size you ordered?" Due to the complexity of the question, an illustration of this pricing structure was provided and explained as part of the question. Less than one-fifth reported noticing this pricing structure (Table 4). The demographic characteristics of those who reported noticing the price modification were similar to those who did not (data not shown).

**Table 4:** Percent of those in the calorie, price, and calories + price experimental conditions who reported noticing the menu modifications

	Calorie^a^ % (n)	Price^b^ % (n)	Calories + price^c^ % (n)
Noticed calories	54.3 (82)	NA^d^	58.7 (88)
Noticed price modification	NA^e^	16.1 (23)	16.7 (25)

^a ^calorie menu included calorie information for each menu item

^b ^price menu had price modification (standardized pricing) for food items with more than one portion size option

^c ^calorie + price menu included calorie information and price modification (standardized pricing) for food items with more than one portion size option.

^d ^Not applicable because price was not modified on the menu

^e ^Not applicable because calories were not included on the menu

To evaluate whether calorie information may have influenced food choices among those who reported noticing this information relative to those who did not, a linear regression analysis was conducted. This analysis was restricted to participants in the calories and calories plus price experimental conditions. Covariates included in the analysis were factors found to differ between those who reported noticing the calorie information and those who did not notice this information, specifically age, race, education level, and site. Results from the multivariate analysis indicated that average energy intake was comparable between those who reported noticing the calorie information and those who did not (690 kcal versus 671 kcal; p = 0.65). Results were similarly null in a comparable analysis conducted to compare energy intake of those who noticed and did not notice the pricing structure modification (p= 0.90).

## Discussion

Results of the present study showed that providing calorie information at the point-of-purchase on a fast food restaurant menu had little effect on food selection and consumption among a sample of adolescents and adults who eat regularly at fast food restaurants. These results contribute to a limited literature on point-of-purchase calorie labeling. To date, seven studies have examined the influence of providing calorie composition information at the point-of-purchase for most food items available in a cafeteria [14,15,17,18,37] or restaurant [24,26] setting. Among these studies, one found no evidence of an effect of calorie labeling on food choices [18]. In contrast, six of the seven studies found some evidence in support of the hypothesis that calorie information may positively influence food choices [14,15,24,17,26], however, results from most of these studies were weak or inconsistent. For example, Conklin et al. found that only 18% of college freshman living on a campus where point-of-purchase nutrition information was available in the dining commons agreed that the available information affected their choice of food [37].

A host of factors may explain the weak and inconsistent results in the literature. Firstly, the calorie labeling formats utilized varied across studies. For example, in one study 5 cm by 5 cm cards with calories in red ink were placed as close as possible to food items in a hospital cafeteria [17]. In contrast, in another cafeteria study calorie information for all menu items was presented on two large posters at the cafeteria entrances, with leaflets distributed to patrons to encourage use of this information [15]. Also, in three studies calorie information was provided along with other nutrient composition information such as saturated fat and fiber [14,24,37]. In the present study, calorie information alone was provided on a restaurant menu in a column between the food item name and price. To draw attention to it, the column was highlighted in bright yellow. Nonetheless, only slightly more than one-half of those who ordered from a menu with calories listed reported noticing this information. The calorie content of meals selected by those who noticed the information compared to those who did not were similar, suggesting our null results are not solely due to the failure of some to notice the calorie information.

The designs of studies conducted to date have varied greatly, with all having limitations. Most notably, four studies measured behavioral intentions [14,24,26,37] rather than actual food choices. Consequently, social desirably bias in reporting is a significant concern in these studies. Other major weaknesses include use of quasi-experimental designs [15,17,18] where factors other than the experimental conditions being tested may have differed across test periods due to lack of randomization. The present study is the first to measure actual food choices within an experimental design where participants were randomly assigned to experimental condition. However, it has methodological weaknesses. Participants were exposed to the calorie information on only one occasion. This is a critical shortcoming if repeated exposure to calorie information is required before awareness or behavior change may be expected.

A final issue is that the weak and inconsistent results across studies may reflect heterogeneity in response to calorie labeling, with certain population subgroups more apt to utilize calorie information when it is provided. For example, a number of studies have found that females are more likely than males to use nutrition information on packaged food products [40-45]. Consequently, it is perhaps not surprising that Milich et al found calorie labeling to effect cafeteria food choices in a sample of female hospital employees [17], whereas Mayer et al. found no significant effect of calorie labeling on cafeteria food choices in a study involving male and females employees of a Fortune 500 company [18]. The results of the present study are consistent with the notion that certain population subgroups may be more likely to use calorie information when it is provided. In the present study males appeared to use the calorie information to choose a higher calorie meal. This finding could be an artifact of multiple comparisons, as a significant number of subgroup analyses were conducted. Conversely, this result could reflect a desire among males for an energy dense meal. To our knowledge, only one other study [24] has reported findings suggesting an unintended consequence of calorie labeling. Yamamoto et al. conducted a study in which adolescents were asked to order meals from three different restaurant menus that did not contain nutrition information, and then reorder their meals after being shown a version of the menus that included calorie and fat content information for menu items. Approximately 17% of meal orders were changed in response to the calorie and fat information. Among the meals that were modified, 20.4% were modified in a way that resulted in a higher calorie content meal.

In the present study the elimination of value size pricing was found to have little influence on food selection or consumption. This finding is somewhat surprising given that a number of studies have documented that price changes may influence food choices [32,46-48]. The price shifts we evaluated tended to be smaller in magnitude compared to those evaluated in previous studies, which could explain why our results conflict with previous findings. It is also possible that the null results are due to the study design which provided only one exposure to the price modification. When queried regarding whether they had noticed the modified pricing structure, less than one-fifth responded affirmatively. Since most fast food restaurant chains utilize a value size price structure, it is possible that study participants generally assumed the larger sized items were the better value without considering the prices listed on the menu. In consideration of this potential methodological issue, future studies designed to evaluate value size pricing should ensure repeated exposure to price modification.

The present study has a number of strengths. The study measured actual food choices rather than behavioral intent. Consequently, social desirability bias in reporting is likely less of a concern and internal validity is probably better than studies that only measured behavioral intent. A randomized design was employed ensuring that potential confounding factors, such as age and sex were equally distributed across experimental conditions. Another strength of the present study is that participants were a community sample of adolescents and adults who ate regularly at fast food restaurants. Consequently, the external validity of results may be stronger than many previous studies.

Limitations of the present study include that participants were exposed to the experimental condition only once. As mentioned earlier, this is problematic as it is possible that repeated exposure to calorie information and standardized pricing may be required before behavior is impacted. Although participants were blinded to the true intent of the study, and the ordering and dining procedure was set-up to be as naturalistic as possible, subject reactivity remains a concern. Of particular concern is the possibility that the participation incentive undermined price sensitivity.

It is important to note that most of the study limitations just described could have been avoided if the study had been conducted in fast food restaurants where menu boards and prices could be manipulated for prolonged periods of time. Unfortunately we were not able to find any fast food restaurants willing to collaborate with us, and thus we were not able to implement a more rigorously designed study.

## Conclusion

In conclusion, results from this study indicate that providing calorie information for food items on fast food restaurant menus may have little effect on the food choices made by adolescents and adults who regularly eat at these establishments. It is possible that skills for using point-of-purchase nutrition information must be built before the information provided may be effectively used. For example, Hawthorne at el. found that knowledge of the basic use of the nutrition facts label on packaged food products was low among a sample of adolescents [49]. After a brief training on use of the label, understanding of the label was significantly improved. More likely though is the need to increase concern about nutrition when eating at fast food restaurants, as factors such as taste and convenience appear to be far more important consideration for most consumers.

Although the design of the present study is more methodologically rigorous than most previous research, it has significant shortcomings. Consequently additional research is warranted to more rigorously evaluate calorie labeling and value size pricing in the context of fast food restaurants. Collaborating with fast food restaurant establishments will be a critical but challenging need.

## Competing interests

The authors declare that they have no competing interests.

## Authors' contributions

LH designed the study and developed the study procedures with input from SF, MS, RJ, MO, and SR. SR coordinated data collection and performed the statistical analysis under the direction of MO and LH. All of the authors were involved in drafting the manuscript. Also, all of the authors read and approved the final manuscript.
